# Functional polymorphisms of the lncRNA *H19* promoter region contribute to the cancer risk and clinical outcomes in advanced colorectal cancer

**DOI:** 10.1186/s12935-019-0895-x

**Published:** 2019-08-20

**Authors:** Wenyan Qin, Xiaodong Wang, Yilin Wang, Yalun Li, Qiuchen Chen, Xiaoyun Hu, Zhikun Wu, Pengfei Zhao, Shanqiong Li, Haishan Zhao, Weifan Yao, Jian Ding, Minjie Wei, Huizhe Wu

**Affiliations:** 1 Department of Pharmacology, School of Pharmacy, Liaoning Key Laboratory of Molecular Targeted Anti-Tumor Drug Development and Evaluation, China Medical University, Shenyang, 110122 People’s Republic of China; 2 Department of Anorectal Surgery, First Hospital of China Medical University, Shenyang, 110001 People’s Republic of China; 3 Division of Antitumor Pharmacology, State Key Laboratory of Drug Research, Shanghai Institute of Materia Medica, Chinese Academy of Sciences, Shanghai, 201203 China

**Keywords:** *H19*, Genetic polymorphisms, Susceptibility, Colorectal cancer, Prognosis

## Abstract

**Background:**

The long non-coding RNA *H19* plays critical roles in cancer occurrence, development, and progression. The present study is for the first time to evaluate the association of genetic variations in the *H19* promoter region with advanced colorectal cancer (CRC) susceptibility, environmental factors, and clinical outcomes.

**Methods:**

16 single-nucleotide polymorphisms (SNPs) were identified in the *H19* gene promoter by DNA sequencing, and 3 SNPs among which including rs4930101, rs11042170, and rs2735970 further expanded samples with 572 advanced CRC patients and 555 healthy controls.

**Results:**

We found that harboring SNP [rs4930101 (*P* = 0.009), rs2735970 (*P* = 0.003), and rs11042170 (*P* = 0.003)] or carrying more than one combined risk genotypes significantly increased the risk for CRC [*P* < 0.0001, adjusted OR (95% CI) 6.48 (2.97–14.15)]. In the correlation analysis with environmental factors, rs2735970 and gender, combined risk genotypes (> 1 vs. ≤ 1) and family history of cancer demonstrated significant interactions. Furthermore, a remarkably worse clinical outcome was found in combined risk genotypes (> 1 vs. ≤ 1), especially in CRC patients with body weight ≥ 61 kg, smoking, and first-degree family history of cancer (Log-rank test: *P* = 0.006, *P* = 0.018, and *P* = 0.013, respectively). More importantly, the multivariate Cox regression analyses further verified that combined risk genotypes > 1 showed a prognostic risk factor for CRC patients with body weight ≥ 61 kg (*P* = 0.002), smoking (*P* = 0.008), and family history of cancer (*P* = 0.006). In addition, MDR analysis consistently revealed that the combination of selected SNPs and nine known risk factors showed a better prediction prognosis and represented the best model to predict advanced CRC prognosis.

**Conclusion:**

3 SNPs of rs4930101, rs11042170, and rs27359703 among 16 identified SNPs of *H19* gene remarkably increased CRC risk. Furthermore, the combined risk genotypes had a significant impact on environmental factors and clinical outcomes in the advanced CRC patients with body weight ≥ 61 kg, ever-smoking, and first-degree family history of cancer. These data suggest that *H19* promoter SNPs, especially these combined SNPs might be more potentially functional biomarkers in the prediction of advanced CRC risk and prognosis.

**Electronic supplementary material:**

The online version of this article (10.1186/s12935-019-0895-x) contains supplementary material, which is available to authorized users.

## Background

Colorectal cancer (CRC) is still the third most commonly occurring cancer both in men and women worldwide. 1.8 million new CRC cases were diagnosed, and 609,000 death cases were reported in 2018 [1]. More importantly, the increased incidence and mortality of CRC were reported in young Asian adults including China [2–4]. The etiology of CRC is complicated in human and multifactor involved in carcinogenesis including environmental exposures, lifestyle factors, and especially multiple inherited genetic variations [5–9]. Non-coding RNA (ncRNAs) is regarded as “a genomic dark matter”, increasing studies have indicated a strong association between single-nucleotide polymorphisms (SNPs) in ncRNAs with the risk for CRC [10–17]. Therefore, to identify genetic variations including those in lncRNA and the interactions between genetic variations with environmental factors could reveal novel diagnostic and prognostic biomarkers for CRC diagnosis and assessments of the treatment accuracy.

Long non-coding RNA (lncRNAs) were first identified in the 1990s [18, 19], which are single-stranded, non-coding RNAs more than 200 nucleotides and no open reading frames (ORF) [20]. Rather than to be transcriptional noise, lncRNAs are the key players with multiple functions in carcinogenesis including regulating cancer cell cycle, proliferation, and apoptosis through regulating gene transcription and posttranscriptional processing [21–24]. The *H19* gene is located on human chromosome 11p15.5, which is a cluster of imprinted genes including H19/insulin like growth factor 2 (IGF2). The *H19* gene encodes 2.3 kb spliced and polyadenylated long noncoding RNA [25–27]. Indeed, H19 is highly expressed in the early stages of embryogenesis, and down-regulated with tissue maturation, however, (re)-expressed in human carcinomas tissues, such as CRC [28–31]. Thus, H19 is involved in cancer initiation, development, and progression, suggesting it could be a critical diagnostic and prognostic biomarker as well as a potential novel target in cancer therapy.

Recent functional studies provide insights into the roles of genetic variants in the *H19* promoter region on the cancer risk, inter-individualized chemotherapy response and prognosis [10, 32–34]. The H19 expression was mainly regulated by *H19* gene upstream 5′-flanking region, which contains differentially methylated regions (DMRs) and mutations [35]. To date, among the more than 100 SNPs found in the *H19* gene (http://www.ncbi.nlm.nih.gov/projects/SNP), some potential functional SNPs in the promoter region play critical roles in altering individual susceptibility to cancer, interaction with environmental factors, and clinical outcomes in CRC [12, 16, 17, 36–39]. Bhatti et al. demonstrated that *H19* rs2107425 polymorphism had close relationships with radiation therapy response in breast cancer patients in the United States (*n* = 859) [40]. O’Brien et al. further recognized that *H19* rs2107425 polymorphism had significantly relationships with breast cancer susceptibility among African–Americans [41]. Yang et al. also reported that the *H19* promoter SNP rs2839698 T allele contributes to the increased gastric cancer risk in a Chinese population [25]. The previous studies focused on *H19* promoter SNP rs2107425 and rs2839698, which are not localized on the high incidence region in the upstream of the *H19* gene. Therefore, to identify potential-functional SNPs in the *H19* promoter region is urgently required which might benefit for early screening initiation and merit investigation.

In this study, we screened the distributions of genetic variation of approximately 3 kb upstream of the *H19* promoter region and further investigated the possible association between every three SNPs in the human *H19* gene (rs4930101, rs11042170, and rs2735970) with advanced CRC risk, environmental factors, and clinical outcomes. Crucially, this study would provide a novel diagnostic biomarker for advanced CRC patients.

## Materials and methods

### Patients and clinical information

This hospital-based case–control study was conducted at China Medical University (Shenyang, China) and approved by the Medical Ethics Committee of China Medical University. Specifically, 572 patients with advanced CRC were recruited from 2008 to 2013 at the First Affiliated Hospital and Shengjing Hospital of China Medical University. The inclusion criteria for CRC patients were: (1) availability of complete clinical data and follow-up status; (2) patients with clinical stage III and IV; and (3) patients underwent FOLFOX6 chemotherapy. The exclusion criteria were: (1) incomplete clinical data; (2) blood samples for genotyping were unavailable; (3) patients only received radiation therapy; (4) patients with other cancers, or cancers with unknown primary sites; (5) patients did not receive the FOLFOX6 regimen. Clinicopathological data were collected including age, gender, first-degree family history of CRC, smoking status, tumor size, tumor differentiation, pathological grade, lymph-node metastases from the interviewer-administered health risk questionnaires and medical records. Non-smokers were defined as individuals who < 100 cigarettes in a lifetime. BMI was calculated from self-reported height and body weight. Tumor differentiation and pathological grade for CRCs were performed according to the World Health Organization criteria. The patients underwent FOLFOX6 regimen for at least 2–3 cycles and were followed up monthly until recurrence or death. Age-, gender-, and ethnicity-matched healthy control volunteers (n = 555) were recruited from the same hospitals. After the interview, 5 ml blood samples were collected for further SNPs genotyping in each group.

### Genotyping

Genomic DNA was extracted from peripheral blood leukocytes using the TIANGEN DNA Blood Mini Kit (TIANGEN Biotech CO., LTD, Beijing, China) and SNP genotyping was performed by TaqMan assay. The probes, primers and the related information about assay conditions, are available upon request. SNP allele-specific probes were labeled with the fluorescent dyes VIC and FAM by using the TaqMan SNP Genotyping Assays on the ABI 7500 Fast Real-Time PCR platform (Applied Biosystems, Life Technologies Corporation, Foster City, CA, USA). The genotyping rates of these SNPs were all above 90%. For quality control, approximately 10% of samples were randomly selected for repeated confirmation. Some of these samples were also confirmed by DNA sequencing analysis. The concordance rate of these repeated samples reached 100%, indicating that the genotyping method and results were reliable.

### Statistical analysis

All data were analyzed via SPSS version 19.0 (SPSS Inc. Chicago, Illinois, USA) and a value of *P* < 0.05 was considered as statistically significant. Correlations between genetic polymorphisms and the susceptibility of CRC and clinical variables were assessed by odds ratios (OR) and 95% confidence intervals (CI) by unconditional logistic regression adjusted for age, gender, body weight, and smoking status. Overall survival (OS) was defined as the time between the surgery and death or last known follow-up. Disease-free survival (DFS) was the time from surgery until recurrence, death, or last known follow-up. Kaplan–Meier curves were used to assess DFS and OS, and the association between the DFS or OS with SNPs was estimated by Log-rank test. Multivariate Cox hazards regression models were used to estimating the adjusted hazard ratios and their 95% CI, thus to evaluate the independent prognostic value of each genotype and clinical variables. The high-order interactions were assessed between the SNPs and clinicopathological parameters by the Multiple Dimension Reduction (MDR) analysis.

## Results

### Identification of SNPs in the promoter region of the *H19* gene

**Fig. 1:**
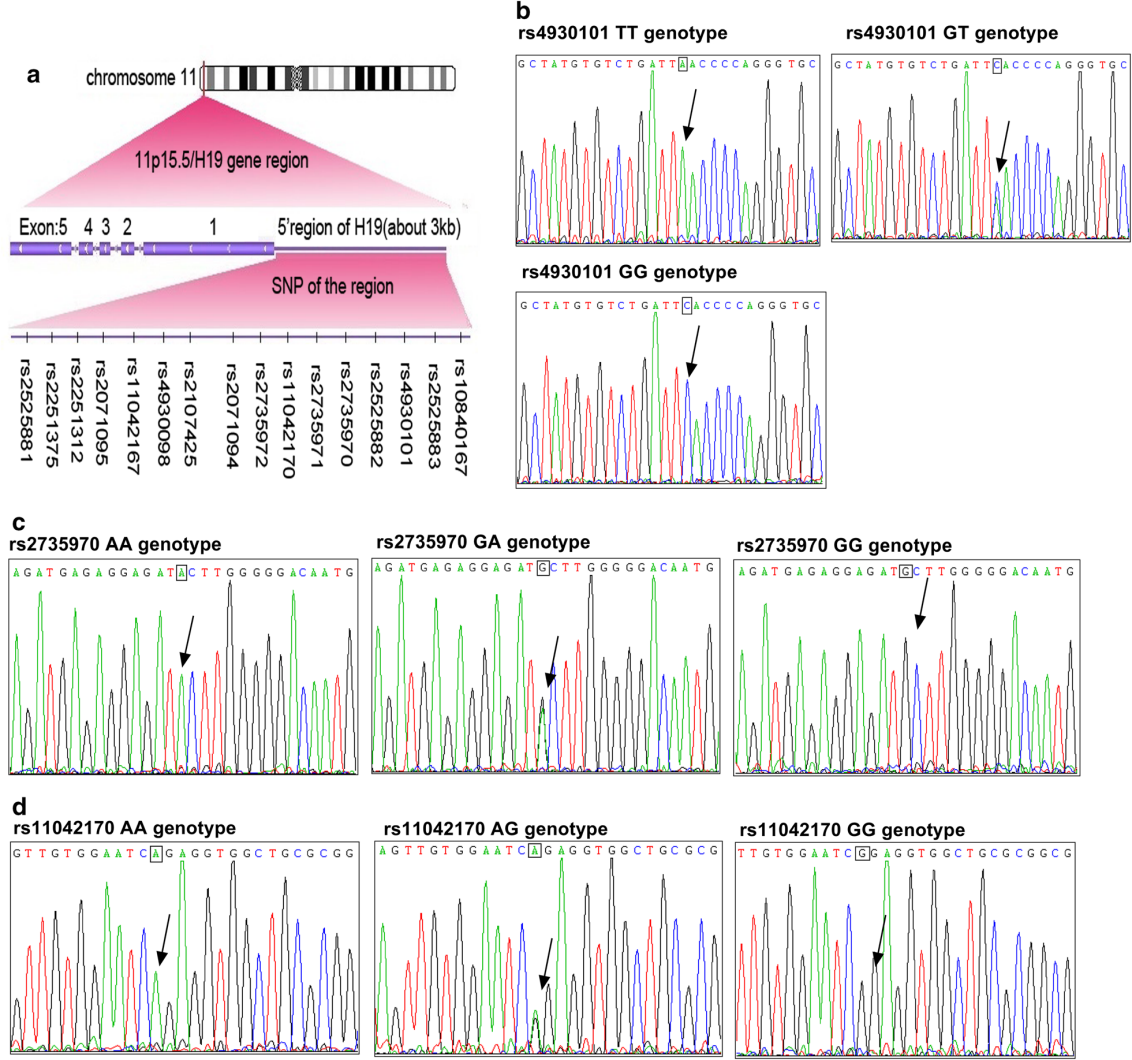
The identified 16 SNPs distribution of about 3 kb upstream of the *H19* promoter region. **a** 16 SNPs distribution in the *H19* promoter region. **b** DNA sequencing genotyping the tagSNPs of rs4930101. **c** DNA sequencing genotyping the tagSNPs of rs2735970, and **d** DNA sequencing genotyping the tagSNPs of rs11042170

To investigate the distribution difference of genetic variants of the *H19* promoter region, the SNPs in approximately 3 kb upstream of *H19* promoter were genotyped in CRC patients (*n* = 51) and healthy controls (*n* = 50) by DNA sequencing. Sixteen SNPs were identified compared with the *Gene Bank* (https://www.ncbi.nlm.nih.gov/snp/), including rs10840167 (G/T), rs2525883 (C/T), rs4930101 (G/T), rs2525882 (T/C), rs2735970 (A/G), rs2735971 (A/G), rs11042170 (G/A), rs2735972 (G/A), rs2071094 (C/A), rs2107425 (C/T), rs4930098 (C/G), rs11042167 (A/G), rs2071095 (G/T), rs2251312 (G/C), rs2251375 (A/C), rs2525881 (T/C) (Additional file 1: Table S1; Fig. 1a). The genotype distributions of those SNPs in the control group were in agreement with the Hardy–Weinberg test (*P* > 0.05, Additional file 1: Table S1). To further evaluate whether those SNPs could affect CRC risk, we carried out a standard allelic association analysis on these SNPs by the *Pearson χ*^*2*^ test and the logistic regression. The frequency distributions of rs4930101 (G/T), rs2735970 (A/G), rs11042170 (G/A) showed significantly different between CRC patients and healthy controls (Additional file 1: Table S1, Fig. 1b–d). Specifically, the SNP rs4930101GG genotype increased the risk for CRC development by 5.211-folds. The combined genotype GT/GG or G allele showed a further significant increase in CRC risk. Harboring rs11042170 GG or GA/GG genotypes suggested a dominant higher risk for CRC development (GG vs. AA: *P* = 0.033, adjusted OR = 5.500, 95% CI 1.027–29.451; GA/GG vs. AA: *P* = 0.034, adjusted OR = 5.067, 95% CI 1.001–25.647, respectively). Moreover, a significantly increased frequency of the rs2735970 AG genotype in CRC patients was observed, compared with that in the healthy controls. In addition, no statistical association was observed between the susceptibility of CRCs and other SNPs of *H19* promoter loci in this cohort (Additional file 1: Table S1).

### The correlation of *H19* rs4930101, rs11042170, rs2735970 with colorectal cancer risk

To study whether *H19* promoter SNPs rs4930101, rs11042170, rs2735970 affect the susceptibility to CRC, we enrolled 572 CRC patients and 555 healthy controls with age and gender-matched. The Median age (range, years) of the CRC group and the control group were 59 (26–82) years and 59 (25–80) years, respectively. There was no statistical difference between the two groups (*P* = 0.789). Demographic data, risk factors and related clinical variables including tumor size, clinical stage, pathological type, lymph node metastasis status, chemotherapy regimen, and other information were list in Additional file 1: Table S2.

**Fig. 2:**
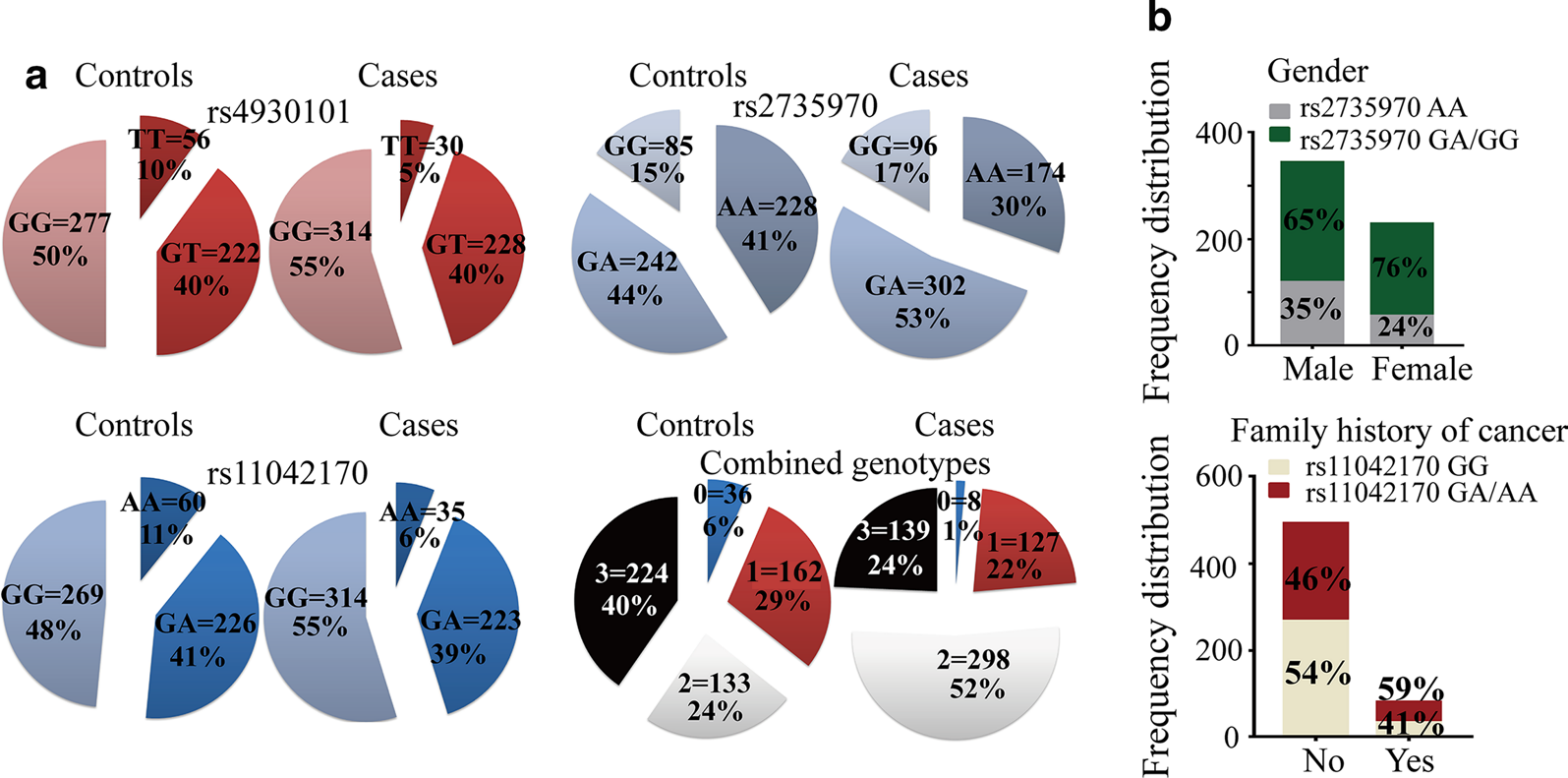
Histogram and box plots illustrating the frequency distribution of rs4930101, rs2735970 and rs11042170 and stratified clinicopathological characteristics. **a** Pie chart illustrating the frequency distribution of rs4930101, rs2735970, and rs11042170 between controls (*n* = 555) and cases (*n* = 572). **b** Histogram chart representing the frequency distribution of rs2735970 genotypes classified by gender (male, female), and rs11042170 genotypes classified by first-degree family history of cancer (no, yes)

By adjusted logistic regression analyses, we found that CRC risk was significantly increased in CRC patients carrying different genotypes of SNP rs4930101, such as heterozygous GT genotype (*P* = 0.007, adjusted OR = 1.92, 95% CI 1.19–3.10), the homozygous GG genotype (*P* = 0.001, adjusted OR = 2.12, 95% CI 1.32–3.39), the dominant model GT/GG genotype (*P* = 0.002, adjusted OR = 2.03, 95% CI 1.28–3.21), and then the G allele (*P* = 0.009, adjusted OR = 1.28, 95% CI 1.06–1.54) (Table 1 and Fig. 2a). SNP rs2735970 was also significantly associated with the increased risk for CRC, such as heterozygous GA genotype (*P* = 0.001, adjusted OR = 1.64, 95% CI 1.26–2.12), the homozygous GG genotype (*P* = 0.029, adjusted OR = 1.48, 95% CI 1.04–2.11) (Table 1 and Fig. 2a). GA/GG genotype (*P* = 0.001, adjusted OR = 1.60, 95% CI 1.25–2.04) and G allele (*P* = 0.003, adjusted OR = 1.29, 95% CI 1.09–1.52) of SNP rs2735970 were also associated with increasing susceptibility of CRCs (Table 1 and Fig. 2a). Moreover, harboring SNP rs11042170 GA, GG genotype, G allele, and GA/GG genotype in dominant model showed significant association with increased CRC risk [GA vs. AA: adjusted OR (95% CI) 1.69 (1.07–2.67), *P* = 0.023; GG vs. AA: adjusted OR (95% CI) 2.00 (1.28–3.13), *P* = 0.002; G vs. A allele: adjusted OR (95% CI) 1.32 (1.09–1.58), *P* = 0.003; and GA/GG vs. AA: 1.86 (1.20–2.87), *P* = 0.005] (Table 1 and Fig. 2a). More importantly, we further elucidated the impact of combined effect of risk genotypes on cancer risk, and found that carrying 1, or 2 or 3 risk genotypes (rs4930101 GT/GG + rs2735970 GA/GG + rs11042170 GA/GG genotype) showed a remarkable increase in the cancer risk [1 risk genotype: *P* = 0.001, adjusted OR (95% CI) 3.53 (1.58–7.86), 2 risk genotypes: *P* < 0.0001, adjusted OR (95% CI) 10.08 (4.56–22.28), 3 risk genotypes: *P* = 0.009, adjusted OR (95% CI) 2.79 (1.26–6.18)] (Table 1 and Fig. 2a). Subsequently, harboring more than 1 risk genotypes of CRC patients significantly increased susceptibility to cancer compared with carrying ≤ 1 risk genotype [*P* < 0.0001, adjusted OR (95% CI) 6.48 (2.97–14.15)] (Table 1 and Fig. 2a). Taken together, these data indicated that the potential function of three SNPs of the *H19* gene is significantly associated with CRC risk.

**Table 1:** Logistic regression analysis of associations between genotypes of *H19* promoter SNPs and advanced CRC susceptibility

Genotypes	Controls number (%)^a^	Cases number (%)	*P* ^b^	Adjusted OR (95% CI)^b^
rs4930101 (G/T)				
TT	56 (10.09)	30 (5.24)		1.00
GT	222 (40.00)	228 (39.86)	*0.007*	*1.92 (1.19–3.10)*
GG	277 (49.91)	314 (54.90)	*0.001*	*2.12 (1.32–3.39)*
GT/GG	499 (89.91)	542 (94.76)	*0.002*	*2.03 (1.28–3.21)*
T allele	334 (30.09)	288 (25.17)		1.00
G allele	776 (69.91)	856 (74.83)	*0.009*	*1.28 (1.06–1.54)*
rs2735970 (G/A)				
AA	228 (41.08)	174 (30.42)		1.00
GA	242 (43.60)	302 (52.80)	*0.001*	*1.64 (1.26–2.12)*
GG	85 (15.32)	96 (16.78)	*0.029*	*1.48 (1.04–2.11)*
GA/GG	327 (58.92)	398 (69.58)	*0.001*	*1.60 (1.25–2.04)*
A allele	698 (62.88)	650 (56.82)		
G allele	412 (37.12)	494 (43.18)	*0.003*	*1.29 (1.09–1.52)*
rs11042170 (G/A)				
AA	60 (10.81)	35 (6.12)		1.00
GA	226 (40.72)	223 (38.99)	*0.023*	*1.69 (1.07–2.67)*
GG	269 (48.47)	314 (54.90)	*0.002*	*2.00 (1.28–3.13)*
GA/GG	495 (89.19)	537 (93.88)	*0.005*	*1.86 (1.20–2.87)*
A allele	346 (31.17)	293 (25.61)		1.00
G allele	764 (68.83)	851 (74.39)	*0.003*	*1.32 (1.09–1.58)*
Combined effect of risk genotypes^c^				
0	36 (6.49)	8 (1.40)		1.00
1	162 (29.19)	127 (22.20)	*0.0012*	*3.53 (1.58–7.86)*
2	133 (23.96)	298 (52.10)	*<* *0.0001*	*10.08 (4.56–22.28)*
3	224 (40.36)	139 (24.30)	*0.0087*	*2.79 (1.26–6.18)*
≤ 1	36 (6.49)	8 (1.40)		1.00
> 1	519 (93.61)	564 (98.60)	*<* *0.0001*	*6.48 (2.97–14.15)*

The significance levels are *P* < 0.05 for all the italics values

^a^The observed genotype frequency among individuals in the control group was in agreement with Hardy–Weinberg equilibrium

^b^*P* values, adjusted OR and 95% CI values were calculated by logistic regression adjusted for age, gender, body weight, smoking status, first-degree family history of cancer status

^c^Risk genotypes used for the calculation were *H19* rs4930101GT/GG + rs2735970GA/GG + rs11042170GA/GG

### The interaction between *H19* promoter SNPs with environmental factors and clinical variables

To explore the clinical utility of the SNP genotypes, the interactive effects of *H19* SNPs between rs4930101, rs11042170, rs2735970 and the environmental factors or clinical variables were determined by *χ*^2^ test and unconditional logistic regression adjusted by gender, ages, smoking status, and first history of cancer (Fig. 2b, Table 2 and Additional file 1: Table S2). We found the significant gender difference in the distribution frequency of *H19* rs2735970 GA/GG genotype [65.4% in man CRC patients, 75.9% in woman CRC patients, *P* = 0.006, the corresponding adjusted OR (95% CI) 1.700 (1.163–2.485)]. The frequency of rs11042170 GA/AA genotype was significantly increased in patients with a family history of cancer (58.8%) compared with those without a family history [45.9%, *P* = 0.035, the corresponding adjusted OR (95% CI) 1.677 (1.038–2.710)] (Fig. 2b and Additional file 1: Table S3). Body weight, smoking and family history of cancer act as the environmental higher risk factors of CRC, we further analyzed the interactions of environmental factors and genetic factors, and identify that combined risk genotypes (> 1 vs. ≤ 1) related to family history of cancer (*P* = 0.028, Table 2).

**Table 2:** Gene-environmental factor interactions (logistic regression)

	rs4930101	rs2735970	rs11042170	Combined genotypes	Weight	Smoking	History
rs4930101		0.055	*<* *0.0001*	*<* *0.0001*	0.154	0.380	0.714
rs2735970	0.055		*<* *0.0001*	*<* *0.0001*	0.102	0.292	0.541
rs11042170	*<* *0.0001*	*0.049*		*<* *0.0001*	0.754	0.099	0.085
Combined genotypes	*<* *0.0001*	*<* *0.0001*	*<* *0.0001*		0.160	0.066	*0.028*
Weight	0.154	0.102	0.754	0.160		0.811	0.793
Smoking	0.380	0.292	0.099	0.066	0.811		*0.010*
History	0.714	0.541	0.085	*0.028*	0.793	*0.010*	

The significance levels are *P* < 0.05 for all the italics values

### Prognostic markers evaluation of *H19* rs4930101, rs11042170, rs2735970 in advanced CRC patients

**Fig. 3:**
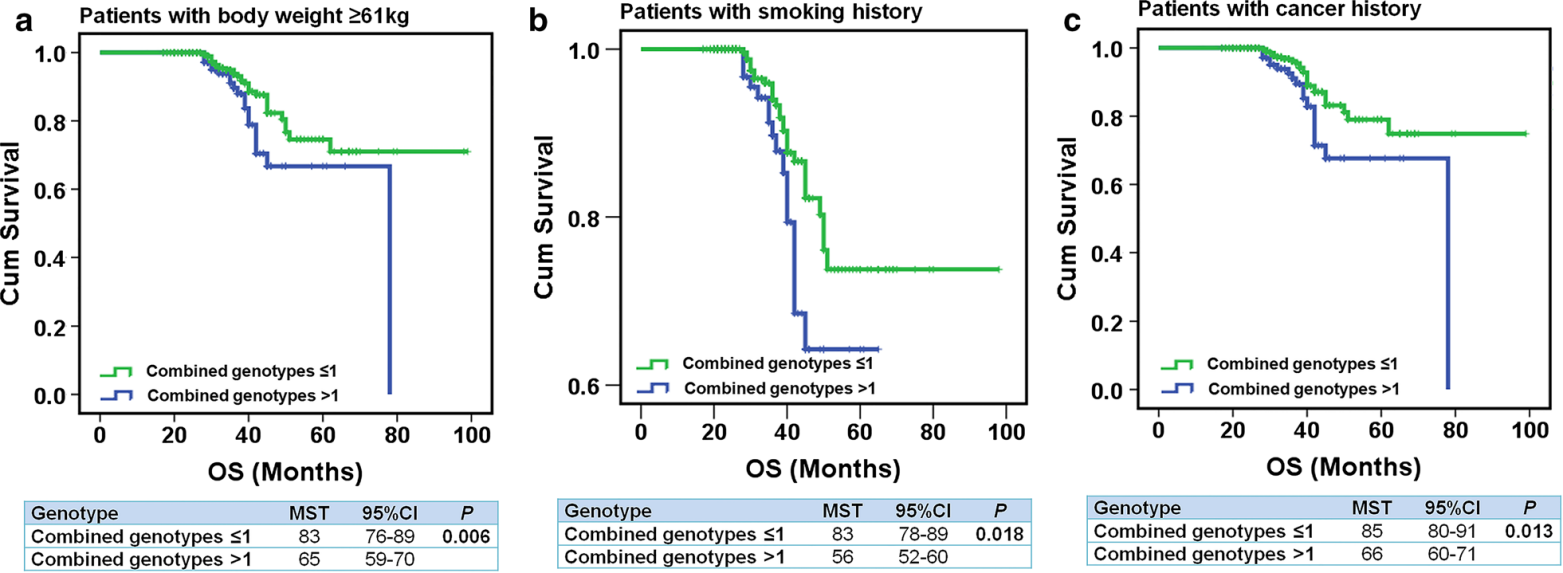
Stratification analysis estimate the correlation of OS and combined genotypes of the *H19* gene in advanced CRC patients using Kaplan–Meier analysis. Stratification analysis illustrating combined genotypes of rs4930101, rs2735970 and rs11042170 (risk genotype > 1) had shorter OS time in advanced CRC patients with body weight ≥ 61 kg (*P* = 0.006) (**a**), smoking history (*P* = 0.018) (**b**), and family history of cancer (*P* = 0.013) (**c**)

To further clarify whether the 3 SNPs of *H19* promoter region were independent prognostic factors in this cohort, we assessed the Log-rank test and multivariate Cox hazard regression analysis including all variables which could affect DFS and OS in CRC patients treated with FOLFOX6 regimen. Overall, there was no statistically significant correlation between the 3 SNPs of the *H19* gene and prognosis. However, remarkably worsen clinical outcomes were found in patients with combined risk genotypes (> 1), especially to those with body weight ≥ 61 kg, smoking, and first-degree family history of cancer (Log-rank test: *P* = 0.006, *P* = 0.018, and *P* = 0.013, respectively) (Fig. 3a–c). The median survival time (MST) in CRC patients with body weight ≥ 61 kg harboring more than 1 combined risk genotypes [MST (95% CI) 65 (59–70) months] was much shorter than those carrying ≤ 1 combined risk genotypes [MST (95% CI) 83 (76–89) months] (Fig. 3a). Meanwhile, in comparison to the reference combined genotypes with the MST on 83 months or 85 months, > 1 combined risk genotype was related to worse overall survival in the patients with smoking [MST (95% CI) 56 (52–60) months] (Fig. 3b) and a family cancer history [MST (95% CI) 66 (60–71) months] (Fig. 3c), respectively. More importantly, the multivariate Cox regression analyses further verified that > 1 combined risk genotypes shows a prognostic risk factor for CRC patients with body weight ≥ 61 kg [*P* = 0.002, HR (95% CI) 1.79 (1.09–2.94)], smoking [*P* = 0.008, HR (95% CI) 2.64 (1.84–3.88)], and a family history of cancer [*P* = 0.006, HR (95% CI) 2.75 (1.17–6.60)] (Table 3).

**Table 3:** Multivariate Cox proportional hazard analyses of *H19* rs4930101, rs2735970, and rs11042170 of in association with DFS and OS in advanced CRC patients

Stratification	DFS			OS		
	Adjusted HR	95% CI^b^	*P* ^a^	Adjusted HR	95% CI^b^	*P* ^a^
Body weight, kg						
rs4930101GG vs. GT/TT	0.97	0.51–1.85	0.924	1.04	0.25–4.35	0.955
rs2375970AA vs. GA/GG	0.87	0.48–1.59	0.658	1.64	0.49–5.56	0.423
rs11042170 AA vs. GA/GG	1.26	0.87–1.83	0.215	1.67	0.81–3.45	0.168
Combined genotypes ≤ 1 vs. > 1	1.18	0.79–1.76	0.421	1.79	1.09–2.94	*0.002*
Smoking						
rs4930101GG vs. GT/TT	0.84	0.53–1.31	0.434	1.56	0.96–2.54	0.073
rs2375970AA vs. GA/GG	1.01	0.62–1.63	0.975	1.40	0.86–2.26	0.075
rs11042170 AA vs. GA/GG	1.33	1.00–1.75	0.047	0.76	0.43–1.32	0.325
Combined genotypes ≤ 1 vs. > 1	2.58	1.89–3.53	*0.001*	2.64	1.84–3.88	*0.008*
First-degree family history of cancer						
rs4930101GG vs. GT/TT	0.82	0.53–1.27	0.368	1.19	0.42–3.32	0.742
rs2375970AA vs. GA/GG	1.02	0.63–1.64	0.952	1.40	0.50–3.92	0.523
rs11042170 AA vs. GA/GG	1.23	0.94–1.62	0.139	1.63	0.90–2.93	0.105
Combined genotypes ≤ 1 vs. > 1	1.22	0.90–1.65	0.203	2.75	1.17–6.60	*0.006*

^a^*P* values were calculated by Multivariate Cox regression analyses adjusted for age, gender, body weight and first-degree family history of cancer status

^b^Adjusted HR and 95% CI values were calculated by Multivariate Cox regression analyses adjusted for age, gender, body weight smoking status and first-degree family history of cancer status

### High-order interactions with CRC prognosis by MDR analysis

To further evaluate the existence of possible gene-environmental factors interaction in association with the clinical outcomes, high-order interactions were assessed by the multiple dimension reduction analysis on the 3 SNPs (rs4930101, rs2735970, and rs11042170), combined genotypes and 8 known risk factors (i.e., age, body weight, gender, smoking status, first-degree family history of cancer, tumor size, tumor differentiation, and clinical stage). In the MDR analysis, 8 risk factors combination was the best model with the highest cross-validation consistency (CVC) and the lowest prediction error in comparison to the one-factor model among all 5 risk factors. The 12-factor model had a maximum CVC and a minimum prediction error, with the prediction error being statistically significant (Table 4) both in DFS and OS. Taken together, the 12-factor model showed a better prediction for prognosis than the 8-factor model and represented the best model to predict CRC prognosis for this study population.

**Table 4:** MDR analysis for the prediction of prognosis with and without 3 SNPs genotypes in advanced CRC patients

Best interaction models	DFS			OS		
	Cross-validation consistency	*P* ^a^	Training odds ratio	Cross-validation consistency	*P* ^a^	Training odds ratio
1	81/100	0.0579	1.38 (0.99–1.92)	83/100	0.0317	1.82 (1.05–3.15)
1,2	65/100	0.0023	1.69 (1.21–2.38)	95/100	0.0032	2.27 (1.30–3.95)
1,2,3	100/100	< 0.0001	2.24 (1.59–3.17)	64/100	0.0001	3.08 (1.69–5.61)
1,2,3,4	100/100	< 0.0001	2.69 (1.91–3.78)	47/100	< 0.0001	4.92 (2.51–9.62)
1,2,3,4,5	100/100	< 0.0001	3.71 (2.61–5.26)	98/100	< 0.0001	14.30 (5.32–38.44)
1,2,3,4,5,6	93/100	< 0.0001	5.08 (3.55–7.27)	90/100	< 0.0001	27.10 (8.79–83.54)
1,2,3,4,5,6,7	61/100	< 0.0001	8.33 (5.63–12.33)	92/100	< 0.0001	71.88 (17.19–300.58)
1,2,3,4,5,6,7,8	100/100	< 0.0001	14.44 (9.40–22.16)	100/100	< 0.0001	∞
1,2,3,4,5,6,7,8,9	54/100	< 0.0001	23.49 (14.80–37.26)	100/100	< 0.0001	∞
1,2,3,4,5,6,7,8,9,10	100/100	< 0.0001	47.27 (27.59–81.01)	100/100	< 0.0001	∞
1,2,3,4,5,6,7,8,9,10,11	100/100	< 0.0001	85.18 (45.85–158.23)	100/100	< 0.0001	∞
*1,2,3,4,5,6,7,8,9,10,11,12*	100/100	< 0.0001	*85.17 (45.84–158.0)*	99/100	< 0.0001	∞

The best model with maximum cross-validation consistency and minimum prediction error rate was in italics

Labels: 1, age, years; 2, body weight; 3, gender; 4, smoking status; 5, first-degree family history of cancer; 6, tumor size (cm); 7, tumor differentiation; 8, clinical stage; 9, rs4930101; 10, rs2735970; 11, rs11042170; 12, combined genotypes

^a^*P* value for 1000-fold permutation test

## Discussion

Although only a small number of lncRNAs have been well-characterized, current studies have revealed that lncRNAs, such as *H19* have been functionally associated with diseases occurrence, development, and progression, in particular, cancers [42, 43]. Dysregulation of lncRNAs has been implicated in breast cancer, bladder cancer, gastric cancer, and colorectal cancer [44–47]. It is evident that dysregulation of *H19* expression affects cellular functions, such as cell proliferation, imprinting, migration, invasion, and metastasis [28, 43, 48–50]. Therefore, the genetic variations of *H19*, especially in the promoter region may play a critical role in affecting the susceptibility to cancer. In the current case–control study with 572 CRC cases and 555 healthy controls from northeast of the Chinese population, for the first time, we explored the potential association between *H19* promoter genetic polymorphisms and CRC risk. We verified that 3 of the 16 included SNPs in the DMR upstream loci of *H19* gene, namely rs4930101, rs11042170, and rs2735970, especially in the combined risk genotypes of the 3 SNPs were remarkably associated with an increased advanced CRC risk, environmental factors, and the clinical outcomes in the advanced CRC patients with body weight ≥ 61 kg, smoking, and first-degree family history of cancer.

In the current study, we first detected the SNPs located at the DMR upstream loci of the *H19* gene in the training set on 51 CRC patients and 50 healthy controls. Total 16 SNPs were identified in this cohort. As the first discovered lncRNA, *H19* is involved in regulating gene expression in the imprinted gene network and contributes to growth control in development [19, 51–54]. Due to the important roles in forensic identification, the 16 SNPs were detected in another two different nationalities, Chinese Han population and Chinese Korean nationality [55, 56], which was consistent with our findings. In this study, because high-quality DNA could be easily prepared from peripheral blood, the genotyping of these SNPs was only identified based on genomic DNA. Van Huis-Tanja et al. [57] reported that 11 SNPs in 9 genes were determined in matched samples from blood and FFPE tissue of colorectal tumors by pyrosequencing and TaqMan techniques. They found only GSTP1 showed significant discordance between FFPE tissue and blood genotype, the discordant rate was only 1.4%. Recently, Shao et al. [58] evaluated the genotyping concordance between tumor tissues and peripheral blood in a genome-wide scale, and high concordant rate (97.42%) was found between tumor tissues and peripheral blood. Thus, we further investigate the relevance of those SNPs with advanced CRC risk and found 3 SNPs among those 16 SNPs showed significantly associated with cancer susceptibility including rs4930101, rs2735970, and rs11042170. With regard to the relationship of the SNPs with CRC risk, we further explored the investigation in a relatively large sample including 572 advanced CRC patients and 555 healthy controls on genomic DNA. Specifically, a significantly increased CRC risk was observed in the advanced CRC patients carrying SNP rs4930101, rs2735970, and rs11042170 homozygous genotype and under the dominant model. More importantly, a remarkably increased 6.48-fold of susceptibility to CRC cancer was determined for the first time in the patients harboring > 1 risk genotypes when compared with carrying ≤ 1 risk genotype (risk genotypes: rs4930101 GT/GG + rs2735970 GA/GG + rs11042170 GA/GG). To our knowledge, it is unclear whether the potential 3 SNPs could affect the expression of *H19* and then develop the cancer risk. However, we found a strong synergistic effect in combined risk genotypes, suggesting they could act as a biomarker in CRC screening and diagnosis.

In this cohort, we further explored the gene-environmental factor interaction of *H19* promoter SNPs rs493010, rs11042170, and rs2735970 with clinicopathological parameters of CRC patients including gender, body weight, smoking and family history of cancer. Although no association was found between rs4930101 and clinical variables, a significantly decreased distribution frequency of rs2735970 AA genotype was observed in the female CRC patients. Importantly, a remarkable relationship was found in the patients who carrying rs11042170 genotype or combined risk genotypes (> 1 vs. ≤ 1) with a family history of cancer. This also indicated that the G allele might be a genetic predisposition factor in advanced CRC. The effect of combined risk genotypes (> 1 vs. ≤ 1) is more significant than the single genotype variation. As cancer is multifactorial, the changes in combined genotypes could dramatically affect cancer development. Recent research found that some variants (rs10505477, rs6983267, rs10795668, and rs11255841) related to CRC risk are associated with the family history of CRC [59]. However, until now, the interaction between those 3 SNPs of *H19* and CRC environmental factors is still unreported. Only one recent case–control study reported another SNP rs2107425 of *H19* promoter region showed a combined greater impact on affecting lung cancer risk than individual effects of the SNPs with cooking smoke exposure [38]. These results indicate that the 3 tag SNPs could serve as potential biomarkers for evaluating the interaction of clinicopathological parameters and advanced CRC associated polymorphisms. Studies on other cancer types and larger sample sizes are encouraged to validate the findings and need to be elucidated and verified in the future.

To further excavate independent prognostic factors in this cohort, we for the first time to perform the log-rank test, multivariate Cox regression analysis, and MDR analysis on all variables to possibly affecting DFS and OS in advanced CRC patients. No significant association was found between *H19* SNPs and CRC overall survival in patients treated with FOLFOX6 regimen. However, the stratification analysis found a remarkably worsen clinical outcomes harboring combined risk genotypes (> 1 vs. ≤ 1) of CRC patients with body weight ≥ 61 kg, smoking, and first-degree family history of cancer, which suggested that combined genotype of the 3 SNPs may affect CRC prognosis and could be a promising biomarker for advanced CRC prognosis. As previously reported, the expression of *H19* could be induced by cigarette smoke and other factors. Therefore, these data suggest that the combined genotypes of the potential SNPs could be functional biomarkers for predicting the prognosis, especially in the CRC patients with specific clinical characteristics including greater body weight, ever-smoking, and first-degree family history of cancer.

In this study, we extensively evaluated the significant associations between SNPs of the *H19* promoter region and CRC risk, pathological features, and clinical outcome in advanced CRC patients for the first time. Our results identified 16 SNPs in the DMR upstream loci of the *H19* gene. The 3 potential SNPs of the rs4930101 G allele, rs11042170 G allele, rs2735970 G allele, and combined risk genotypes were associated with increased advanced CRC risk in a training set and overall cohort. Furthermore, interactions of those SNPs and combined risk genotypes with environmental factors, and prognosis were found in the advanced CRC patients with body weight ≥ 61 kg, smoking, and first-degree family history of cancer. However, functional experiments are warranted to further elucidate the role of *H19* and the underlying molecular mechanism in CRC tumorigenesis.

## Conclusions

1. 3 SNPs of rs4930101, rs11042170, and rs27359703 among 16 identified SNPs in the DMR upstream loci of the *H19* gene were remarkably associated with an increased risk for advanced CRC.
2. CRC patients who are harboring > 1 combined risk genotypes showed a remarkably increased CRC risk (6.48-fold) and a significant interaction with environmental factors.
3. It is notable that a significantly worse impact on clinical outcomes was observed in the stratification analysis, especially in the CRC patients harboring combined risk genotypes (> 1 vs. ≤ 1) with body weight ≥ 61 kg, ever-smoking, and first-degree family history of cancer.
4. Future in vitro and in vivo studies in patients with other cancers are needed to confirm these findings.

## Additional file

**Additional file 1.** Additional tables.

## Data Availability

The datasets used and/or analyzed during the current study are available from the corresponding author on reasonable request.

## Abbreviations

CRC: colorectal cancer
ncRNA: non-coding RNA
DMRs: differentially methylated regions
OR: odds rations
CI: confidence intervals
OS: overall survival
DFS: disease-free survival
MDR analysis: multiple dimension reduction analysis

## References

[1] Siegel RL, Miller KD, Jemal A (2018). Cancer statistics, 2018. CA Cancer J Clin.

[2] Chen W, Zheng R, Baade PD, Zhang S, Zeng H, Bray F (2016). Cancer statistics in China, 2015. CA Cancer J Clin.

[3] Tsoi KKF, Hirai HW, Chan FCH, Griffiths S, Sung JJY (2017). Predicted increases in incidence of colorectal cancer in developed and developing regions, in association with ageing populations. Clin Gastroenterol Hepatol..

[4] Connell LC, Mota JM, Braghiroli MI, Hoff PM (2017). The rising incidence of younger patients with colorectal cancer: questions about screening, biology, and treatment. Curr Treat Options Oncol.

[5] Broderick P, Dobbins SE, Chubb D, Kinnersley B, Dunlop MG, Tomlinson I (2017). Validation of recently proposed colorectal cancer susceptibility gene variants in an analysis of families and patients—a systematic review. Gastroenterology..

[6] Lochhead P, Chan AT, Giovannucci E, Fuchs CS, Wu K, Nishihara R (2014). Progress and opportunities in molecular pathological epidemiology of colorectal premalignant lesions. Am J Gastroenterol.

[7] Park CH, Eun CS, Han DS (2018). Intestinal microbiota, chronic inflammation, and colorectal cancer. Intest Res..

[8] Abu-Remaileh M, Bender S, Raddatz G, Ansari I, Cohen D, Gutekunst J (2015). Chronic inflammation induces a novel epigenetic program that is conserved in intestinal adenomas and in colorectal cancer. Cancer Res.

[9] Zhang K, Civan J, Mukherjee S, Patel F, Yang H (2014). Genetic variations in colorectal cancer risk and clinical outcome. World J Gastroenterol.

[10] Gao P, Wei GH (2017). Genomic insight into the role of lncRNA in cancer susceptibility. Int J Mol Sci..

[11] Evans JR, Feng FY, Chinnaiyan AM (2016). The bright side of dark matter: lncRNAs in cancer. J Clin Invest..

[12] Xu B, Zhu Y, Tang Y, Zhang Z, Wen Q (2018). Rs4938723 polymorphism is associated with susceptibility to hepatocellular carcinoma risk and is a protective factor in leukemia, colorectal, and esophageal cancer. Med Sci Monit.

[13] Hua JT, Ahmed M, Guo H, Zhang Y, Chen S, Soares F (2018). Risk SNP-mediated promoter-enhancer switching drives prostate cancer through lncRNA PCAT19. Cell..

[14] Xia W, Zhu XW, Mo XB, Wu LF, Wu J, Guo YF (2017). Integrative multi-omics analysis revealed SNP-lncRNA–mRNA (SLM) networks in human peripheral blood mononuclear cells. Hum Genet.

[15] Zhang X, Zhou L, Fu G, Sun F, Shi J, Wei J (2014). The identification of an ESCC susceptibility SNP rs920778 that regulates the expression of lncRNA HOTAIR via a novel intronic enhancer. Carcinogenesis.

[16] Li Z, Niu Y (2018). Association between lncRNA H19 (rs217727, rs2735971 and rs3024270) polymorphisms and the risk of bladder cancer in Chinese population. Minerva Urol Nefrol..

[17] Li L, Guo G, Zhang H, Zhou B, Bai L, Chen H (2018). Association between H19 SNP rs217727 and lung cancer risk in a Chinese population: a case control study. BMC Med Genet.

[18] Brannan CI, Dees EC, Ingram RS, Tilghman SM (1990). The product of the H19 gene may function as an RNA. Mol Cell Biol.

[19] Jarroux J, Morillon A, Pinskaya M (2017). History, discovery, and classification of lncRNAs. Adv Exp Med Biol.

[20] Jia H, Osak M, Bogu GK, Stanton LW, Johnson R, Lipovich L (2010). Genome-wide computational identification and manual annotation of human long noncoding RNA genes. RNA.

[21] Kopp F, Mendell JT (2018). Functional classification and experimental dissection of long noncoding RNAs. Cell.

[22] Wang J, Xu W, He Y, Xia Q, Liu S (2018). LncRNA MEG3 impacts proliferation, invasion, and migration of ovarian cancer cells through regulating PTEN. Inflamm Res..

[23] Zhao C, Wang S, Zhao Y, Du F, Wang W, Lv P (2018). Long noncoding RNA NEAT1 modulates cell proliferation and apoptosis by regulating miR-23a-3p/SMC1A in acute myeloid leukemia. J Cell Physiol..

[24] Lei Q, Pan Q, Li N, Zhou Z, Zhang J, He X (2018). H19 regulates the proliferation of bovine male germline stem cells via IGF-1 signaling pathway. J Cell Physiol..

[25] Yang C, Tang R, Ma X, Wang Y, Luo D, Xu Z (2015). Tag SNPs in long non-coding RNA H19 contribute to susceptibility to gastric cancer in the Chinese Han population. Oncotarget..

[26] Coto E, Diaz Corte C, Tranche S, Gomez J, Reguero JR, Alonso B (2018). Genetic variation in the H19-IGF2 cluster might confer risk of developing impaired renal function. DNA Cell Biol.

[27] Cui P, Zhao Y, Chu X, He N, Zheng H, Han J (2018). SNP rs2071095 in LincRNA H19 is associated with breast cancer risk. Breast Cancer Res Treat.

[28] Raveh E, Matouk IJ, Gilon M, Hochberg A (2015). The H19 Long non-coding RNA in cancer initiation, progression and metastasis—a proposed unifying theory. Mol Cancer..

[29] Ding D, Li C, Zhao T, Li D, Yang L, Zhang B (2018). LncRNA H19/miR-29b-3p/PGRN axis promoted epithelial–mesenchymal transition of colorectal cancer cells by acting on Wnt signaling. Mol Cells.

[30] Matsuzaki H, Okamura E, Takahashi T, Ushiki A, Nakamura T, Nakano T (2015). De novo DNA methylation through the 5′-segment of the H19 ICR maintains its imprint during early embryogenesis. Development..

[31] Ariel I, de Groot N, Hochberg A (2000). Imprinted H19 gene expression in embryogenesis and human cancer: the oncofetal connection. Am J Med Genet.

[32] Lavie O, Edelman D, Levy T, Fishman A, Hubert A, Segev Y (2017). A phase 1/2a, dose-escalation, safety, pharmacokinetic, and preliminary efficacy study of intraperitoneal administration of BC-819 (H19-DTA) in subjects with recurrent ovarian/peritoneal cancer. Arch Gynecol Obstet.

[33] Chu M, Yuan W, Wu S, Wang Z, Mao L, Tian T (2016). Quantitative assessment of polymorphisms in H19 lncRNA and cancer risk: a meta-analysis of 13,392 cases and 18,893 controls. Oncotarget..

[34] Dugimont T, Montpellier C, Adriaenssens E, Lottin S, Dumont L, Iotsova V (1998). The H19 TATA-less promoter is efficiently repressed by wild-type tumor suppressor gene product p53. Oncogene.

[35] Do EK, Zucker NL, Huang ZY, Schechter JC, Kollins SH, Maguire RL (2018). Associations between imprinted gene differentially methylated regions, appetitive traits and body mass index in children. Pediatr Obes..

[36] Li S, Hua Y, Jin J, Wang H, Du M, Zhu L (2016). Association of genetic variants in lncRNA H19 with risk of colorectal cancer in a Chinese population. Oncotarget..

[37] Wu Q, Yan W, Han R, Yang J, Yuan J, Ji X (2016). Polymorphisms in long noncoding RNA H19 contribute to the protective effects of coal workers’ pneumoconiosis in a Chinese population. Int J Environ Res Public Health..

[38] Yin Z, Cui Z, Li H, Li J, Zhou B (2018). Polymorphisms in the H19 gene and the risk of lung Cancer among female never smokers in Shenyang, China. BMC Cancer..

[39] Guo QY, Wang H, Wang Y (2017). LncRNA H19 polymorphisms associated with the risk of OSCC in Chinese population. Eur Rev Med Pharmacol Sci..

[40] Bhatti P, Doody MM, Alexander BH, Yuenger J, Simon SL, Weinstock RM (2008). Breast cancer risk polymorphisms and interaction with ionizing radiation among U.S. radiologic technologists. Cancer Epidemiol Biomarkers Prev..

[41] O’Brien KM, Cole SR, Poole C, Bensen JT, Herring AH, Engel LS (2014). Replication of breast cancer susceptibility loci in whites and African Americans using a Bayesian approach. Am J Epidemiol.

[42] Li CF, Li YC, Wang Y, Sun LB (2018). The effect of LncRNA H19/miR-194-5p axis on the epithelial–mesenchymal transition of colorectal adenocarcinoma. Cell Physiol Biochem.

[43] Li JP, Xiang Y, Fan LJ, Yao A, Li H, Liao XH (2018). Long noncoding RNA H19 competitively binds miR-93-5p to regulate STAT3 expression in breast cancer. J Cell Biochem..

[44] Jiang M, Xiao Y, Liu D, Luo N, Gao Q, Guan Y (2018). Overexpression of long noncoding RNA LINC01296 indicates an unfavorable prognosis and promotes tumorigenesis in breast cancer. Gene.

[45] Liu Z, Xie D, Zhang H (2018). Long noncoding RNA neuroblastoma-associated transcript 1 gene inhibits malignant cellular phenotypes of bladder cancer through miR-21/SOCS6 axis. Cell Death Dis..

[46] Zhang E, He X, Zhang C, Su J, Lu X, Si X (2018). A novel long noncoding RNA HOXC-AS3 mediates tumorigenesis of gastric cancer by binding to YBX1. Genome Biol.

[47] Xu M, Chen X, Lin K, Zeng K, Liu X, Pan B (2018). The long noncoding RNA SNHG1 regulates colorectal cancer cell growth through interactions with EZH2 and miR-154-5p. Mol Cancer..

[48] Yoshimura H, Matsuda Y, Yamamoto M, Michishita M, Takahashi K, Sasaki N (2018). Reduced expression of the H19 long non-coding RNA inhibits pancreatic cancer metastasis. Lab Invest.

[49] Tserga A, Binder AM, Michels KB (2017). Impact of folic acid intake during pregnancy on genomic imprinting of IGF2/H19 and 1-carbon metabolism. FASEB J..

[50] Rokavec M, Horst D, Hermeking H (2017). Cellular model of colon cancer progression reveals signatures of mRNAs, miRNA, lncRNAs, and epigenetic modifications associated with metastasis. Cancer Res.

[51] Yoshimura H, Matsuda Y, Yamamoto M, Kamiya S, Ishiwata T (2018). Expression and role of long non-coding RNA H19 in carcinogenesis. Front Biosci (Landmark Ed)..

[52] Ghazal S, McKinnon B, Zhou J, Mueller M, Men Y, Yang L (2015). H19 lncRNA alters stromal cell growth via IGF signaling in the endometrium of women with endometriosis. EMBO Mol Med..

[53] Murphy R, Thompson JM, Tost J, Mitchell EA, Auckland Birthweight Collaborative Study G (2014). No evidence for copy number and methylation variation in H19 and KCNQ10T1 imprinting control regions in children born small for gestational age. BMC Med Genet..

[54] Monnier P, Martinet C, Pontis J, Stancheva I, Ait-Si-Ali S, Dandolo L (2013). H19 lncRNA controls gene expression of the Imprinted Gene Network by recruiting MBD1. Proc Natl Acad Sci USA..

[55] Wei WT, Wang X, Wang DM, Tian LY, Wang BJ, Pang H (2013). SNP in differentially methylated region upstream of H19 gene in Chinese Korean nationality. Fa Yi Xue Za Zhi..

[56] Ma XY, He WZ, Yuan TL, Xiao JJ, Wang XM, Li SY (2016). SNP in differentially methylated region upstream of H19 gene in Guangdong Han population. Fa Yi Xue Za Zhi..

[57] van Huis-Tanja L, Kweekel D, Gelderblom H, Koopman M, Punt K, Guchelaar HJ (2013). Concordance of genotype for polymorphisms in DNA isolated from peripheral blood and colorectal cancer tumor samples. Pharmacogenomics..

[58] Shao W, Ge Y, Ma G, Du M, Chu H, Qiang F (2017). Evaluation of genome-wide genotyping concordance between tumor tissues and peripheral blood. Genomics.

[59] Gargallo CJ, Lanas A, Carrera-Lasfuentes P, Ferrandez A, Quintero E, Carrillo M (2019). Genetic susceptibility in the development of colorectal adenomas according to family history of colorectal cancer. Int J Cancer.

