# Supplementary material for: Functional polymorphisms of the lncRNA H19 promoter region contribute to the cancer risk and clinical outcomes in advanced colorectal cancer
Source: Cancer Cell Int. 2019 Aug 20;19:215. doi: 10.1186/s12935-019-0895-x (PMC6702740; doi:10.1186/s12935-019-0895-x)
Supplement: Supplementary file 1 — Additional file 1. Additional tables. [file 12935_2019_895_MOESM1_ESM.docx]

**Functional polymorphisms of lncRNA *H19* promoter region contribute to the risk and clinical outcomes in advanced colorectal cancer**

Wenyan Qin^1,4^, Xiaodong Wang^1,4^,Yilin Wang^1^, Yalun Li^2^, Qiuchen Chen^1^, Xiaoyun Hu^1^, Zhikun Wu^1^, Pengfei Zhao^1^, Shanqiong Li^1^, Haishan Zhao^1^, Weifan Yao^1^, Jian Ding^1,3^*, Minjie Wei^1^*,and Huizhe Wu^1^*

**Outline**

- **Additional data**
- **Table S1** Frequency distribution of the polymorphisms in the promoter region of *H19* gene and their associations with the risk of developing advanced colorectal cancer
- **Table S2** Clinical characteristics of cases (*n*=572) and controls (*n*=555)
- **Table S3** Correlations of *H19* polymorphisms with environmental factor and clinical parameters in advanced colorectal cancer patients

**Table S1 Frequency distribution of the polymorphisms in the promoter region of *H19* gene and their associations with the risk of developing advanced colorectal cancer**

| Genotypes | Controls  Number(%) | Cases  number(%) | *P* ^a^ | Adjusted OR(95%CI)^b^ | HWE^c^ |
| --- | --- | --- | --- | --- | --- |
| rs10840167 (G/T ) | *n*=38 | *n*=34 |  |  |  |
| GG | 19(50.00) | 18 (52.94) |  | 1 (Reference) | 0.066 |
| GT | 12(31.58) | 9(26.47) | 0.671 | 0.792(0.269-2.327) |  |
| TT | 7(18.42) | 7(20.59) | 0.931 | 1.056(0.308-3.612) |  |
| GT/TT | 19(50.00) | 16(47.06) | 0.803 | 0.889(0.352-2.244) |  |
| rs2525883 (C/T) | *n*=37 | *n*=37 |  |  |  |
| CC | 20(54.05) | 18(48.65) |  | 1 (Reference) | 0.804 |
| CT | 14(37.84) | 16(43.24) | 0.625 | 1.270(0.487-3.314) |  |
| TT | 3 (8.11) | 3(8.11) | 0.905 | 1.111(0.198-6.220) |  |
| CT/TT | 17(45.95) | 19(51.35) | 0.642 | 1.242(0.498-3.094) |  |
| C | 54(72.97) | 52(70.27) |  | 1 (Reference) |  |
| T | 20(27.03) | 22(29.73) | 0.715 | 1.142(0.559-2.336) |  |
| rs4930101 (G/T) | *n*=44 | *n*=38 |  |  |  |
| TT | 9(20.46) | 2(5.27) |  | 1 (Reference) | 0.122 |
| GT | 16(36.36) | 14(36.84) | 0.098 | 3.938(0.735-21.377) |  |
| GG | 19(43.18) | 22 (57.89) | **0.036** | **5.211(1.000-27.146)** |  |
| GT/GG | 35(77.78) | 36(94.74) | **0.044** | **4.629(0.933-22.954)** |  |
| T | 34(38.64） | 18(23.68） |  | 1 (Reference) |  |
| G | 54(61.36） | 58(76.32） | **0.040** | **2.029(1.027-4.009)** |  |
| rs2525882 (T/C) | *n*=37 | *n*=36 |  |  |  |
| TT | 19(51.35) | 16 (44.44) |  | 1 (Reference) | 0.986 |
| CT | 15(40.54) | 19(52.78) | 0.398 | 1.504(0.582-3.885) |  |
| CC | 3(8.11) | 1(2.78) | 0.429 | 0.396(0.037-4.187) |  |
| CT/CC | 18(48.65) | 20(55.56) | 0.555 | 1.319(0.525-3.313) |  |
| T | 53(71.62） | 51(70.83） |  | 1 (Reference) |  |
| C | 21(28.38） | 21(29.17） | 0.916 | 1.039(0.508-2.128) |  |
| rs2735970 (A/G) | *n*=37 | *n*=42 |  |  |  |
| AA | 14(37.84) | 7 (18.92) |  | 1 (Reference) | 0.531 |
| AG | 16(43.24) | 25(67.57) | **0.039** | 3.125(1.037-9.418) |  |
| GG | 7(18.92) | 5(13.51) | 0.632 | 1.429(0.331-6.170) |  |
| AG/GG | 23(62.16) | 35(81.08) | 0.071 | 2.609(0.906-7.511) |  |
| A | 44(59.46） | 39(52.70） |  | 1 (Reference) |  |
| G | 30(40.54） | 35(47.30） | 0.408 | 1.316(0.687-2.523) |  |
| rs2735971 (A/G) | *n*=37 | *n*=37 |  |  |  |
| AA | 0(0.00) | 1 (2.70) |  | 1 (Reference) | 0.400 |
| AG | 9(24.32) | 13(35.14) | 0.412 | 0.474(0.017-12.926) |  |
| GG | 28(75.68) | 23(62.16) | 0.275 | 0.275(0.011-7.066) |  |
| AG/GG | 37(100.10) | 36(97.30) | 0.314 | 0.324(0.013-8.226) |  |
| A | 9(12.16） | 15(20.27） |  | 1 (Reference) |  |
| G | 65(87.84） | 59(79.73） | 0.181 | 0.545(0.222-1.377) |  |
| rs11042170 (G/A) | *n*=38 | *n*=40 |  |  |  |
| AA | 8 (21.05) | 2(5.00) |  | 1 (Reference) | 0.158 |
| GA | 14(36.84) | 16(40.00) | 0.067 | 4.571(0.829-25.211) |  |
| GG | 16(42.11) | 22(55.00) | **0.033** | **5.500(1.027-29.451)** |  |
| GA/GG | 30(78.95) | 38(95.00) | **0.034** | **5.067(1.001-25.647)** |  |
| A | 30(39.47) | 20(29.90) |  | 1 (Reference) |  |
| G | 46(60.53) | 60(70.10) | 0.052 | 1.957(0.987-3.877) |  |
| rs2735972 (G/A) | *n*=37 | *n*=37 |  |  |  |
| CC | 31(83.75) | 35(94.59) |  | 1 (Reference) | 0.199 |
| CT | 5(13.51) | 2(5.41) | 0.218 | 0.354(0.064-1.958) |  |
| TT | 1 (2.7) | 0(0.00) | 0.292 | 0.296(0.012-7.525) |  |
| CT/TT | 6(16.21) | 2(5.41) | 0.134 | 0.295(0.055-1.571) |  |
| C | 67(90.54) | 72(97.29) |  | 1 (Reference) |  |
| T | 7(9.45) | 2(2.71) | 0.166 | 0.266(0.053-1.325) |  |
| rs2071094 (C/A) | *n*=33 | *n*=36 |  |  |  |
| CC | 18(54.55) | 21(58.33) |  | 1 (Reference) | 0.632 |
| AC | 12(36.36) | 13(36.11) | 0.885 | 0.929(0.340-2.539) |  |
| AA | 3 (9.09) | 2(5.56) | 0.560 | 0.571(0.086-3.808) |  |
| AC/AA | 15(45.45) | 15(41.67) | 0.751 | 1.857(0.330-2.223) |  |
| C | 48(72.73) | 55(76.39) |  | 1 (Reference) |  |
| A | 18(27.27) | 17(23.61) | 0.621 | 0.824(0.383-1.776) |  |
| rs2107425 (C/T) | *n*=42 | *n*=47 |  |  |  |
| CC | 14(33.33) | 14(29.79) |  | 1 (Reference) | 0.126 |
| CT | 16(38.10) | 22(46.81) | 0.524 | 1.375(0.515-3.669) |  |
| TT | 12 (28.57) | 11(23.40) | 0.877 | 0.917(0.304-2.764) |  |
| CT/TT | 28(66.67) | 33(70.21) | 0.719 | 1.179(0.481-2.887) |  |
| C | 44(52.38) | 50(53.19) |  | 1 (Reference) |  |
| T | 40(47.62) | 44(46.81) | 0.914 | 0.968(0.537-1.745) |  |
| rs4930098 (C/G) | *n*=36 | *n*=38 |  |  |  |
| CC | 22(61.11) | 26(54.17) |  | 1 (Reference) | 0.120 |
| CG | 10(27.78) | 19(39.58) | 0.327 | 1.608(0.620-4.171) |  |
| GG | 4 (11.11) | 3(6.25) | 0.576 | 0.635(0.128-3.146) |  |
| CG/GG | 14(38.89) | 22(45.83) | 0.524 | 1.330(0.552-3.200) |  |
| C | 54(75.00) | 71(73.96) |  | 1 (Reference) |  |
| G | 18(25.00) | 25(26.04) | 0.878 | 1.056(0.524-2.131) |  |
| rs11042167 (A/G) | *n*=44 | *n*=51 |  |  |  |
| AA | 4(9.09) | 9(17.65) |  | 1 (Reference) | 0.138 |
| GA | 12(27.27) | 19(37.25) | 0.617 | 0.704(0.177-2.802) |  |
| GG | 28 (63.64) | 23(45.10) | 0.120 | 0.365(0.099-1.340) |  |
| GA/GG | 40(90.91) | 42(82.35) | 0.226 | 0.467(0.133-1.637) |  |
| A | 20(22.73) | 37(36.27) |  | 1 (Reference) |  |
| G | 68(77.27) | 65(63.73) | 0.420 | 0.517(0.272-0.981) |  |
| rs2071095 (G/T) | *n*=44 | *n*=49 |  |  |  |
| GG | 28(63.64) | 23(46.94) |  | 1 (Reference) | 0.187 |
| GT | 12(27.27) | 23(46.94) | 0.060 | 2.333(0.959-5.680) |  |
| TT | 4(9.09) | 3(6.12) | 0.911 | 0.913(0.185-4.501) |  |
| GT/TT | 16(36.36) | 26(53.06) | 0.106 | 1.978(0.861-4.545) |  |
| G | 68(77.27） | 69(70.41） |  | 1 (Reference) |  |
| T | 20(22.73） | 29(29.59） | 0.289 | 1.429(0.738-2.767) |  |
| rs2251312 (G/C) | *n*=50 | *n*=38 |  |  |  |
| GG | 30(60.00) | 25(65.79) |  | 1 (Reference) | 0.729 |
| GC | 18(36.00) | 12(31.58) | 0.628 | 0.800(0.324-1.973) |  |
| CC | 2 (4.00) | 1(2.63) | 0.681 | 0.600(0.051-7.012) |  |
| GC/CC | 20(40.00) | 13(34.21) | 0.578 | 0.780(0.324-1.875) |  |
| G | 78(78.00) | 62(81.58) |  | 1 (Reference) |  |
| C | 22(22.00) | 14(18.42) | 0.560 | 0.801(0.379-1.692) |  |
| rs2251375 (A/C) | *n*=37 | *n*=38 |  |  |  |
| CC | 10 (27.02) | 9(23.68) |  | 1 (Reference) | 0.147 |
| AC | 14(37.84) | 23(60.53) | 0.289 | 1.825(0.596-5.590) |  |
| AA | 13(35.14) | 6(15.79) | 0.319 | 0.513(0.137-1.923) |  |
| AC/AA | 27(72.97) | 29(76.32) | 0.739 | 1.193(0.421-3.383) |  |
| C | 34(45.95) | 41(53.95) |  | 1 (Reference) |  |
| A | 40(54.05) | 35(46.05) | 0.327 | 0.726(0.382-1.379) |  |
| rs2525881(T/C) | *n*=28 | *n*=37 |  |  |  |
| TT | 17(60.71) | 18(48.65) |  | 1 (Reference) | 0.208 |
| TC | 8(28.57) | 17(45.95) | 0.199 | 2.007(0.688-5.853) |  |
| CC | 3(10.72) | 2(5.40) | 0.633 | 0.630(0.093-4.244) |  |
| TC/CC | 11(39.29) | 19(51.35) | 0.334 | 1.631(0.603-4.414) |  |
| T | 42(75.00) | 53(71.62) |  | 1 (Reference) |  |
| C | 14(25.00) | 21(28.38) | 0.667 | 1.189(0.540-2.614) |  |

The significance levels are *P*<0.05 for all the bold values.

^a^*P* values were calculated from 2-sided chi-square tests for either genotype distribution or allele frequency.

^b^Adjusted OR and 95% CI values were calculated by unconditional logistic regression adjusted for age, gender, body weight, smoking status and first-degree family history of cancer status.

^c^HWE, the observed genotype frequency among individuals in the control group agreed with Hardy-Weinberg equilibrium.

**Table S2 Clinical characteristics of cases (*n*=572) and controls (*n*=555)**

| Characteristic | Cases |  | Controls |  | *P* |
| --- | --- | --- | --- | --- | --- |
|  | **Number** | **%** | **Number** | **%** |  |
| Total number | 572 | 100 | 555 | 100 |  |
| Median age (range), yrs | 59 (26-82) |  | 59(25-80) |  |  |
| Mean body weight (SD), kg | 61(8.56) |  | 69(8.45) |  |  |
| Age at diagnosis, yrs |  |  |  |  |  |
| <59 | 277 | 48.43 | 273 | 49.19 | 0.789 |
| ≥59 | 295 | 51.57 | 282 | 50.81 |  |
| Body weight, kg |  |  |  |  |  |
| <61 | 314 | 54.90 | 78 | 14.05 | **0.001** |
| ≥61 | 258 | 45.10 | 477 | 85.95 |  |
| Gender |  |  |  |  |  |
| Male | 344 | 60.14 | 353 | 64.69 | 0.339 |
| Female | 228 | 39.86 | 202 | 35.31 |  |
| First-degree family history of cancer |  |  |  |  |  |
| No | 492 | 86.01 | 492 | 88.65 | 0.849 |
| Yes | 80 | 13.99 | 63 | 11.35 |  |
| Smoking |  |  |  |  |  |
| Never^a^ | 479 | 83.74 | 535 | 96.40 | **0.001** |
| Ever | 93 | 16.26 | 20 | 3.60 |  |
| Tumor size (cm) |  |  |  |  |  |
| <6cm | 347 | 60.67 |  |  |  |
| ≥6cm | 225 | 39.33 |  |  |  |
| Tumor differentiation |  |  |  |  |  |
| Grade 1 (G1, Well) | 46 | 8.04 |  |  |  |
| Grade 2 (G2, moderate) | 469 | 81.99 |  |  |  |
| Grade 3 (G3, poor) | 57 | 9.97 |  |  |  |
| Clinical stages |  |  |  |  |  |
| Stage III | 267 | 57.17 |  |  |  |
| Stage IV | 245 | 42.83 |  |  |  |
| Lymph node metastases |  |  |  |  |  |
| Negative | 226 | 39.51 |  |  |  |
| Positive | 346 | 60.49 |  |  |  |

^a^Deﬁned as <100 cigarettes in lifetime.

The significance levels are *P*< 0.05 for all the bold values.

**Table S3 Correlations of *H19* polymorphisms with environmental factor and clinical parameters in advanced colorectal cancer patients**

| Characteristic | rs4930101 | | |  | rs2735970 | | |  | rs11042170 | | |  |
| --- | --- | --- | --- | --- | --- | --- | --- | --- | --- | --- | --- | --- |
|  | **TT**  ***n*(%)** | **GT/GG**  ***n*(%)** | ***P***^a^**^,b^** | **Adjusted OR(95%CI) ^c^** | **AA**  ***n*(%)** | **GA/GG**  ***n*(%)** | ***P***^a,b^ | **Adjusted OR(95%CI) ^c^** | **GG**  ***n*(%)** | **GA/AA**  ***n*(%)** | ***P***^a,b^ | **Adjusted OR(95%CI) ^c^** |
| Age, yrs |  |  |  |  |  |  |  |  |  |  |  |  |
| <60 | 26(9.4) | 251(90.6) | 0.497 | 1.000(Reference) | 76(27.4) | 201(72.6) | 0.133 | 1.000(Reference) | 148(53.4) | 129(46.6) | 0.591 | 1.000(Reference) |
| ≥60 | 23(7.8) | 272(92.2) | 0.453 | 1.254(0.694-2.266) | 98(33.2) | 197(66.8) | 0.079 | 0.722(0.502-1.039) | 151(51.2) | 144(48.8) | 0.707 | 1.066(0.764-1.486) |
| Body weight, kg |  |  |  |  |  |  |  |  |  |  |  |  |
| <61 | 29(9.2) | 285(90.8) | 0.528 | 1.000(Reference) | 92(29.3) | 222(70.7) | 0.521 | 1.000(Reference) | 166(52.9) | 148(47.1) | 0.754 | 1.000(Reference) |
| ≥61 | 20(7.8) | 238(92.2) | 0.502 | 1.228(0.675-2.233) | 82(31.8) | 176(68.2) | 0.329 | 0.835(0.580-1.200) | 133(51.6) | 125(48.4) | 0.704 | 1.067(0.764-1.489) |
| Gender |  |  |  |  |  |  |  |  |  |  |  |  |
| Male | 30(8.7) | 314(91.3) | 0.871 | 1.000(Reference) | 119(34.6) | 225(65.4) | **0.008** | 1.000(Reference) | 178(51.7) | 166(48.3) | 0.756 | 1.000(Reference) |
| Female | 19(8.3) | 209(91.7) | 0.930 | 1.028(0.561-1.880) | 55(24.1) | 173(75.9) | **0.006** | **1.700(1.163-2.485)** | 121(53.1) | 107(46.9) | 0.645 | 0.923(0.658-1.296) |
| Smoking |  |  |  |  |  |  |  |  |  |  |  |  |
| Never | 40(8.4) | 439(91.6) | 0.676 | 1.000(Reference) | 150(31.3) | 329(68.7) | 0.291 | 1.000(Reference) | 258(53.9) | 221(46.1) | 0.084 | 1.000(Reference) |
| Ever | 9(9.7) | 84(90.3) | 0.571 | 0.801(0.372-1.727) | 24(25.8) | 69(74.2) | 0.268 | 1.336(0.800-2.230) | 41(44.1) | 52(55.9) | 0.139 | 1.407(0.895-2.214) |
| First-degree family history of cancer | | | |  |  |  |  |  |  |  |  |  |
| No | 43(8.7) | 449(91.3) | 0.713 | 1.000(Reference) | 152(30.9) | 340(69.1) | 0.541 | 1.000(Reference) | 266(54.1) | 226(45.9) | **0.033** | 1.000(Reference) |
| Yes | 6(7.5) | 74(92.5) | 0.690 | 1.200(0.490-2.936) | 22(27.5) | 58(72.5) | 0.662 | 1.127(0.659-1.928) | 33(41.3) | 47(58.8) | **0.035** | **1.677(1.038-2.710)** |
| Tumor size (cm) |  |  |  |  |  |  |  |  |  |  |  |  |
| ≤6.0 | 10(7.4) | 126(92.6) | 0.562 | 1.000(Reference) | 37(27.2) | 99(72.8) | 0.351 | 1.000(Reference) | 71(52.2) | 65(47.8) | 0.986 | 1.000(Reference) |
| >6.0 | 39(8.9) | 397(91.1) | 0.551 | 0.802(0.389-1.655) | 137(31.4) | 299(68.6) | 0.323 | 0.804(0.521-1.239) | 228(52.3) | 208(47.7) | 0.981 | 0.995(0.675-1.467) |
| Tumor differentiation | |  |  |  |  |  |  |  |  |  |  |  |
| Grade 1 | 4(8.7) | 42(91.3) | 0.908 | 1.000(Reference) | 17(37.0) | 29(63.0) | 0.502 | 1.000(Reference) | 29(63.0) | 17(37.0) | 0.232 | 1.000(Reference) |
| Grade 2 | 41(8.7) | 428(91.3) | 0.768 | 1.245(0.291-5.333) | 138(29.4) | 331(70.6) | 0.714 | 1.169(0.508-2.691) | 238(50.7) | 231(49.3) | 0.626 | 1.223(0.544-2.749) |
| Grade 3 | 4(7.0) | 53(93.0) |  |  | 19(33.3) | 38(66.7) | 0.419 | 0.779(0.426-1.426) | 32(56.1) | 25(43.9) | 0.225 | 0.703(0.397-1.243) |
| Clinical stage |  |  |  |  |  |  |  |  |  |  |  |  |
| III | 34(10.4) | 293(89.6) | 0.071 | 1.000(Reference) | 101(30.9) | 226(69.1) | 0.779 | 1.000(Reference) | 181(55.4) | 146(44.6) | 0.089 | 1.000(Reference) |
| IV | 15(6.1) | 230(93.9) | 0.608 | 1.328(0.449-3.927) | 73(29.8) | 172(70.2) | 0.808 | 1.046(0.726-1.507) | 118(48.2) | 127(51.8) | 0.072 | 1.360(0.973-1.901) |

^a^P values were calculated from 2-sided chi-square tests or Fisher’s Exact Test.

^b^*P* values were calculated by unconditional logistic regression adjusted for age, gender, body weight and first-degree family history of cancer status.

^c^Adjusted OR and 95% CI values were calculated by unconditional logistic regression adjusted for age, gender, body weight, smoking status and first-degree family history of cancer status.
